# Applicability of an In-Vitro Digestion Model to Assess the Bioaccessibility of Phenolic Compounds from Olive-Related Products

**DOI:** 10.3390/molecules26216667

**Published:** 2021-11-03

**Authors:** Patricia Reboredo-Rodríguez, Carmen González-Barreiro, Elena Martínez-Carballo, Noelia Cambeiro-Pérez, Raquel Rial-Otero, María Figueiredo-González, Beatriz Cancho-Grande

**Affiliations:** Food and Health Omics, Department of Analytical and Food Chemistry, Faculty of Science, University of Vigo, 32004-Ourense, Spain; preboredo@uvigo.es (P.R.-R.); cargb@uvigo.es (C.G.-B.); elena.martinez@uvigo.es (E.M.-C.); ncambeiro@uvigo.es (N.C.-P.); raquelrial@uvigo.es (R.R.-O.); bcancho@uvigo.es (B.C.-G.)

**Keywords:** olive oil, table olives, olive byproducts, phenolic compounds, in-vitro digestion, bioaccessibility, bioavailability

## Abstract

The Mediterranean diet includes virgin olive oil (VOO) as the main fat and olives as snacks. In addition to providing nutritional and organoleptic properties, VOO and the fruits (olives) contain an extensive number of bioactive compounds, mainly phenolic compounds, which are considered to be powerful antioxidants. Furthermore, olive byproducts, such as olive leaves, olive pomace, and olive mill wastewater, considered also as rich sources of phenolic compounds, are now valorized due to being mainly applied in the pharmaceutical and nutraceutical industries. The digestive system must physically and chemically break down these ingested olive-related products to release their phenolic compounds, which will be further metabolized to be used by the human organism. The first purpose of this review is to provide an overview of the current status of in-vitro static digestion models for olive-related products. In this sense, the in-vitro gastrointestinal digestion methods are widely used with the following aims: (i) to study how phenolic compounds are released from their matrices and to identify structural changes of phenolic compounds after the digestion of olive fruits and oils and (ii) to support the functional value of olive leaves and byproducts generated in the olive industry by assessing their health properties before and after the gastrointestinal process. The second purpose of this review is to survey and discuss all the results available to date.

## 1. Literature Search Strategy

All the information used for the preparation of this review was found in three electronic databases: Scopus, Web of Science (WoS), and PubMed. The following string was used for all, and the terms were applied to specific parts of the documents (in particular, title, abstract, or keywords): (evoo OR “extra virgin olive oil” OR “olive oil” OR olive) AND (phenol* OR polyphenol*) AND (bioavailab* OR bioaccessib*) AND (digesti*). No limit was established on the search period or in terms of language.

A total of 45 publications were found in WoS, of which 33 were found in Scopus, and 17 were found in PubMed. Duplicate publications were excluded, and the resulting ones were scanned to retrieve additional relevant research. After this search, the final number of articles selected for review was 23 original studies that fit with the aim of this review: evaluating the bioaccessibility of phenolic compounds from olive-related products after simulated gastrointestinal in-vitro digestion. Among these, nine of the reviewed articles used olive oil as the matrix; four articles used olive fruits; five articles used olive leaves; four articles used olive oil byproducts; and finally, one article used commercial standards.

## 2. Simulated Gastrointestinal In-Vitro Digestion Methodologies

Olive farming is an important agricultural activity in the European Union’s (EU’s) southern member states. Around 4.6 million ha were harvested in 2017. Spain, with 2.53 million ha, has the largest area under cultivation, followed by Italy (1.06 million ha), Greece (0.69 million ha), and Portugal (0.32 million ha) [1]. The other olive-producing member states—France, Croatia, Cyprus, and Slovenia—together accounted for 1% of the EU’s total olive tree area [1]. The EU dominates the international olive oil market. Therefore, the olive tree (*Olea europaea* L.) plays a crucial role in Mediterranean countries not only for olive oil production but also for table olives. 

The Mediterranean diet (MedDiet) is characterized by a high intake of phenolic compounds, which are present in the main foods of this dietary pattern: virgin olive oil (VOO, the main lipid source), nuts, red wine, legumes, vegetables, fruits and whole-grain cereals [2]. Phenolic compounds are important candidates responsible for the beneficial effects of the MedDiet. Daily intake of phenolic compounds is very important because of their associated health benefits. Higher total consumption than 600 mg per day provides a protective effect against chronic diseases [3,4]. It is important to know that not only the quantities but also the chemical structures of these compounds are closely related to the positive biological actions in the diet [5]. 

**Figure 1:**
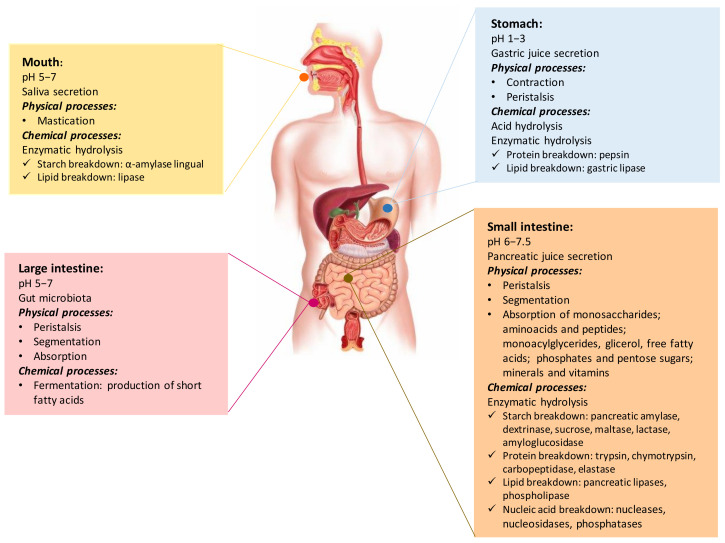
Physical and chemical processes occurring in the human digestive system.

To understand the physiological response to phenolic compounds of foods, it is necessary to follow the digestive processes within the human digestive tract (see Figure 1). Briefly, the oral phase involves food introduction through the mouth, which is broken down into small pieces while mixed with saliva; exposure of the food to salivary amylase and lingual lipase splits starch and triglycerides, respectively. The oral bolus is transferred to the stomach by peristaltic contractions of the esophagus and diluted with the gastric juice, which contains water, hydrochloric acid, electrolytes (sodium, potassium, calcium, phosphate, sulfate, and bicarbonate), mucus, enzymes (lipase and pepsinogen), hormones (gastrin, serotonin), and the intrinsic factor [6,7]. Gastric pH ranges from 1–3 (in the fasted state) to 5.5–7 (after the ingestion of a meal); it changes to 4–5 (after the half emptying time), and it turns again to its basal value (after the stomach emptied) [8]. The acidic conditions are responsible for acid hydrolysis of food; meanwhile, pepsin and gastric lipase are responsible for the protein and lipid breakdown, respectively. Gastric contractions allow to mix food particles with digestive juice and reduce particle sizes to form chyme [6,7]. Enzymatic hydrolysis and absorption processes in the small intestine, facilitated by pancreatin enzymes (proteases—trypsin and chymotrypsin, pancreatic lipase, and pancreatic amylase) and bile (bile salts, phospholipids, cholesterol, bilirubin electrolytes, and water), allow to convert chyme into suitable forms to be absorbed [6,7]. The final breakdown of digestion products happens on the surface of the brush border, which secretes enzymes (maltase-glucoamylase, sucrase-isomaltase, lactase, brush border peptidases, lipase) that hydrolyze disaccharides, peptides, and nucleotides to their basic units suitable for absorption, such as monosaccharides and amino acids; these monomers are absorbed by the intestinal wall and transported to the bloodstream [6,7]. The large intestine is colonized by a gut microbiota that produces enzymes capable of breakdown of dietary fibers, metabolization of bile salts and pancreatic enzymes that reach the colon, and synthesis of vitamins; free fatty acids are considered as byproducts of the gut microbiota fermentation [7].

Digestion of food in the human digestive system is a complex combination of several physicochemical processes (described in Figure 1) that control food intake, disintegration to suitable forms, absorption of the basic units, transportation to related organs, and purging the remaining waste [7]. Since the food matrix affects the release of phenolic compounds and their transport to the target sites during digestion, it is essential to understand the underlying mechanisms affecting their release during digestion for health benefit [7].

Bioavailability is defined as the proportion of a particular compound that is digested, absorbed, metabolized, and used in the organism for normal bodily functions. Bioavailability of phenolic compounds from food samples depends on their release from the food matrix and the structural changes that can be produced during the gastrointestinal digestion (bioaccessibility) [5,9,10] as well as the cellular uptake of glycoside forms of phenolic compounds and respective aglycons by enterocytes, fermentation of nonabsorbed phenolic compounds by gut microbiota and formation and absorption of metabolites, and the metabolism of all absorbed compounds [11]. To determine the bioaccessibility and bioavailability of phenolic compounds, in-vivo (human or animal) intervention trials can be considered [12]. In-vivo trials simulate digestion better, but they are complex because they depend on host-related factors (intestinal factors, gender, age, disorders, or physiological conditions); they are expensive and lengthy, and they are not justifiable on ethical grounds [7,12,13,14]. 

For these reasons, the static in-vitro methods have been used as a suitable, inexpensive, and simple alternative to assess the bioaccessibility of particular compounds from the food matrix under gastrointestinal conditions at the endpoint. They are relatively inexpensive and technically simple. In the static in-vitro digestion models, food samples are subjected to sequential oral, gastric, and intestinal digestion; these models do not usually take the large intestine into account because the absorption of compounds mainly takes place in the small intestine [15]. The static methods use a constant ratio of food to enzymes, electrolytes, and a constant pH for each phase. They can obtain proper simulation data for the understanding of the digestibility and stability of food, release from the food matrix, and structural changes of bioactive compounds after the digestion process [5,10,16,17,18]. Moreover, cell culture models, such as Caco-2 and HepG2 cells, in combination with in-vitro digestion facilitate the study of small intestinal absorption and metabolism and thus elucidate the potential impacts of these compounds on human health [10].

Different studies published from 2012 to 2021 (23 in total) and summarized in Table 1, are focused on the use of in-vitro digestion to study the relationship between the bioaccessibility of phenolic compounds of *Olea oleuropa* L. matrices—olive fruits (17%), olive oils (39%), olive leaves (22%) and other olive byproducts (17%)—and their potential health benefits. These methodologies aim to simulate the physiological appropriateness conditions of the upper gastrointestinal tract to guarantee (i) the reproducible release and the sufficient amount of enzymes required for hydrophilic phenolic compound cleavage and cellular uptake and (ii) the separation of the bioaccessible phase from the unavailable matrix.

As can be seen in Table 1, the in-vitro digestion methodologies mainly differed from one another in the number and type of steps included in the digestion sequence (e.g., mouth, stomach, small intestine), in the composition of the digestive fluids used in each step (e.g., enzymes, salts, buffers), and in the experimental conditions that may affect enzyme activities (e.g., ratios of enzyme to substrate, pH, temperature, or duration of digestive phase). This fact may have affected the results, making a comparison between studies very difficult. Currently, there is a standard protocol for the digestion of food matrices and the evaluation of micronutrients and/or phytochemicals release from the food matrix. This protocol, “INFOGEST static in-vitro simulation of gastrointestinal digestion” (SGD) [19], is based on an international consensus developed by the COST INFOGEST network. It was one of the aims of the INFOGEST network to not only standardize in-vitro methods but to agree on experimental conditions based on available physiological data that are as close as possible to the in-vivo equivalent [19]. To date, this protocol has only been applied to assess the bioaccessibility of the phenolic compounds from VOO [20] and olive pomace extracts (OPEs) [11].

Of all these factors, the most important one in an in-vitro digestion system is the enzyme characteristics: the composition and concentration of enzymes. Most enzymes considered for in-vitro digestion studies are collected or extracted from omnivorous animals, i.e., pigs, rats, or human volunteers. With respect to the enzyme composition, single-enzyme methods can be useful for predicting the digestibility of single nutrients (e.g., protein by the use of pepsin, starch by the use of amylase, or lipids by the use of lipase) [21,22], and they are often advantageous because the standardization of in-vitro digestion models is facilitated, which enables more consistent laboratory-to-laboratory comparisons [22,23]. However, the types of enzymes included within an in-vitro digestion model tend to reflect the major food components being investigated, so the enzyme composition of a particular digestive fluid can often be simulated by mixing together appropriate amounts of pure enzymes [21,22]. Concerning the enzyme concentration, higher enzyme concentrations accelerate digestion or degradation of food components, and therefore, it is important to use physiologically relevant levels.

The addition of lipases is useful for predicting the digestibility of lipids. Lipases are present in the stomach (gastric lipase) and pancreas (pancreatic lipase), where they are absorbed to the surfaces of emulsified lipids and convert triacylglycerols and diacylglycerols to monoacylglycerols and free fatty acids. As is shown in Table 1, the pH conditions ranged from 2 to 3, and porcine pepsin is the only enzyme added in the gastric digestion, except in the study developed by Reboredo et al., who also added gastric lipase [20]. Lipid digestion starts in the stomach with the action of preduodenal lipase (gastric lipase in humans) on triacylglycerides (TAGs) and some other esters. Gastric lipolysis not only contributes to the overall digestion of TAGs but also triggers the subsequent action of pancreatic lipase on lipid substrates that may be poorly digested by pancreatic lipase alone. It is therefore recommended to add gastric lipase during the gastric phase of in-vitro digestion; rabbit gastric lipase is an acceptable substitute for human gastric lipase and is now commercially available. To stop pepsin activity, the pH of gastric samples is raised to 6.5–8.1 by adding NaOH or NaHCO_3_; with the addition of pancreatin in the small intestine digestion, bovine bile is preferred because its composition is similar to that of human bile [19]. The final lipid digestion products in the small intestine are those solubilized within mixed micelles and vesicles that transport them to the epithelium cells through the mucous layer.

The composition of simulated salivary (SSF), gastric (SGF), or intestinal fluids (SIF) is essential because enzymes often require additional components within these digestive fluids to operate efficiently [21,22]. As an example, the activity of pancreatic lipase depends on the presence of co-lipase, bile salts, and calcium [22,24]. Other authors reported that calcium reacts with liberated free fatty acids by means of ionic complexation, thereby removing them from the surface of the lipid droplets and preventing them from inhibiting the lipase [22,25]. The use of the following salts—KCl, KH_2_PO_4_, NaHCO_3_, NaCl, MgCl_2_(H_2_O)_6_, (NH_4_)_2_CO_3_, HCl, and CaCl_2_(H_2_O)_2_—in aqueous fluids adjusted at different pHs is recommended by the standardized INFOGEST protocol [19]. When Čepo et al. [11] assessed the bioaccessibility of OPEs, they considered SSF and SIF according to INFOGEST protocol, fed-state-simulated fluids (FeSSGF and FeSSIF), and fasted-state-simulated fluids (FaSSGF and FASSIF). The composition of three media does not influence the bioaccessibility of hydroxytyrosol (HTy) and tyrosol (Ty) from olive pomace because there were no differences among results; fed- and fasted-state media can slightly improve the bioaccessibility of other phenolic compounds.

All in-vitro digestion models surveyed in this review considered 37 °C as the digestion temperature despite the variations in the enzymes employed. The length of the incubation times of the samples in the simulated digestive fluids should mimic the reported digestion times in humans. With regard to digestion time, it depends in-vivo upon individual characteristics. In practice, a range of digestion times from 20 min to 2 h has been reported for incubation of samples in simulated stomach, meanwhile, 2 h has been predominantly employed for simulated in the small intestine (Table 1). It is worth emphasizing that a short transit time of food within the small intestine may limit the absorption of bioactive lipophilic compounds, thereby reducing their bioavailability [22,26].

Food matrix effects on bioaccessibility of phenolic compounds from olive pomace were recently studied by Čepo et al. [11]. OPEs were co-digested with different foods according to INFOGEST protocol. Soy, milk formula, cheese, and breakfast cereals exerted negative effects on HTy and Ty bioaccessibility, probably due to negative HTy/Ty-casein and HTy/Ty-dietary fiber interactions.

## 3. Olive Oils

Consumers are taking greater responsibility for their own health, and they are increasingly focusing upon their diet to improve it. The MedDiet is characterized by high consumption of exogenous dietary phenolic compounds as a consequence of high intakes of VOO, fruits, nuts, vegetables, and cereals; moderate intakes of fish and poultry; low intakes of dairy products, red meat, processed meats, and sweets; and wine in moderation, consumed with meals [42]. Among these foods, VOO stands out (as the main source of fat) [5,31,34] not only for its nutritional and organoleptic properties but also because its consumption provides great health benefits [33,43], which makes it unique and characteristic with respect to other vegetable oils [34,44].

The health-promoting effects of VOO have been ascribed not only to monounsaturated fatty acids, mostly oleic acid (C18:1 n-9), but also phenolic compounds [34,45]. On average, VOO consists of 400–500 mg/kg of phenolic compounds. Phenolic compounds are mainly responsible for their antioxidant capacity (AC) [10], and they have been gaining special interest because many studies have shown their strong biological effects—anti-inflammatory, anti-cancer, anti-atherogenic, and hypoglycemic; additionally, they are able to prevent coronary and degenerative processes [17,33,43]. Among this phenolic group, Ty, HTy, oleuropein (Ole), oleocanthal, and flavonoids stand out due to their anti-inflammatory and anti-teratogenic activity and by improving the lipid profile, reducing oxidative stress, activating inflammatory cells, and generally protecting against oxidative damage [17,33,46].

Scientific evidence of protection against oxidative stress was provided to the Panel of the European Food Safety Agency (EFSA) with the results of the EUROLIVE study. In the list of health claims that have been made about foods, as referred to in Article 13 (3) of Regulation (EC) No 1924/2006 [47], a health claim has been established for olive oil phenolic compounds. The claim is that “olive oil polyphenols contribute to the protection of blood lipids from oxidative stress”. The condition of use of the claim is that it “may be used only for olive oils which contain at least 5 mg of HTy and its derivatives (e.g., oleuropein complex and Ty) per 20 mg of olive oil”. In order to bear the claim, information shall be given to the consumer that the beneficial effect is obtained with a daily intake of 20 mg of olive oil.

The effect of the digestion of olive oil on the bioaccessibility of its phenolic compounds has been studied far more than the same topic with any other olive matrices. To date, it should be noted that ultraviolet-visible (UV-VIS) methods, such as Folin–Ciocalteu and DPPH methods, have been the most used in the assessment of the bioaccessibility of phenolic compounds from VOO (as is described in Table 2 and Table 3); notwithstanding, more specific information about changes in phenolic profiles after SGD conditions have been obtained by chromatographic techniques.

### 3.1. Assessment of the Total Phenolics in Oils and Their Bioaccessibility by Using UV-VIS Methods

#### 3.1.1. Effect of Olive Variety on the Initial Phenolic Composition of VOO

Although the composition and concentrations of phenolic compounds in VOO depend on environmental factors (soil and climate), agronomic factors (irrigation, fertilization), cultivation (harvesting and ripeness), and technological questions (post-harvest storage and extraction system), the different varieties of olive fruits are mainly responsible for the different phenolic profiles of VOO [31,48,49]. Authors whose experimental results are listed in Table 2 and Table 3 highlighted the effect of olive variety using the Folin–Ciocalteu and DPPH procedures. It should be pointed out that UV-VIS methods are simple, rapid, inexpensive, and non-specific assays widely applied for the determination of total phenolic content (TPC) or AC. Moreover, the use of different standards for quantification (Ty, caffeic acid equivalents (CAE), gallic acid equivalents (GA)) makes it difficult to compare the results.

Dinnella et al. [5] determined TPC by Folin–Ciocalteu of ten Italian extra virgin olive oils (EVOOs) (EVOO 1–EVOO 10, Table 2) from three different varieties (Coratina, Maiatica, and Oliarola del Bradano). The TPC ranged from 277 mg Ty/kg for EVOO 5 (Maiatica) to 713 mg Ty/kg for EVOO 9 (Coratina). A similar study was performed by Borges et al. [10], who determined the TPC of six Spanish monovarietal EVOO made from Arbequina (EVOO 16), Cornicabra (EVOO 11), Hojiblanca (EVOO 15), Manzanilla (EVOO 13), Picual (EVOO 12), and Picudo (EVOO 14) varieties. The TPC (mg CAE/kg) were as follows: Cornicabra (317) > Picual (256) > Manzanilla (234) > Picudo (207) > Hojiblanca (169) > Arbequina (153); significant differences (*p* < 0.05) between these monovarietal oils were observed (Table 2). It has been reported that Cornicabra and Picual are the Spanish olive oils with the highest TPC. With respect to the AC measured by the DPPH method, Cornicabra, Manzanilla, and Picudo oils showed the highest values, as can be seen in Table 3. Seiquer et al. [12] determined higher values in Picual EVOO (EVOO 17, Table 3).

Borges et al. [17] studied how the fruits of one variety, Arbequina, cultivated in different Brazilian and Spanish regions, can have different phenolic contents. Samples from eleven different geographical areas from Spain (EVOO 18–EVOO 26) and Brazil (EVOO 27 and EVOO 28) were studied. The TPC of Arbequina oils presented a large range from 75 to 302 mg CAE/kg. EVOO 28 (Minas Gerais, Brazil) and EVOO 20 (Málaga, Spain) had the lowest and the highest values, respectively (see Table 2). The EVOOs from Málaga and Valladolid presented the highest AC (1.58 and 1.52 mmol Trolox/kg, respectively (see Table 3)). It has been shown that the geographic and climatic conditions of production areas may affect the quality and composition of olive oils [50]. It was found that the maximum temperature of the growing zone was positively related (*p* < 0.001) with the TPC (*r* = 0.801) and with the antioxidant properties of the oil extracts (DPPH, *r* = 0.715), whereas rainfall correlated negatively with TPC (*p* < 0.01, *r* = 0.520). In accordance, Brazilian oils, produced in regions with high levels of rainfall, showed on average lower values of TPC than Spanish oils (see Table 2). No global differences in AC were found among oils from different countries (Table 3).

Finally, Borges et al. [32] assessed the composition and antioxidant properties of two Spanish EVOOs from cultivars Hojiblanca and Arbequina, regarding harvest year and crop stage. The TPC was higher in Hojiblanca (367–405 mg CAE/kg) than in Arbequina (222–231 mg CAE/kg) EVOOs for all harvest and crop stages, and a general increase in phenolic compounds was observed from early to late oils in the same harvest period. Thus, it was necessary to determine the best ripening stage for each variety in order to obtain high-quality olive oils. However, the measured antioxidant markers varied greatly depending on the year of harvest and the crop stage, unlike what was observed for TPC (Table 3). These findings support the antioxidant quality of EVOOs being partly attributable to compounds other than phenolic compounds. In fact, the authors proposed that high levels of coenzyme Q10 (CoQ10) of Arbequina (EVOO 35) have a positive role in its antioxidant power, as CoQ10 is an electron acceptor and a potent antioxidant.

#### 3.1.2. Bioaccessible Fractions

As it was said above, the bioaccessible fraction corresponds to the phenolic fraction released from the olive oil matrix in the gastrointestinal tract, which then becomes available for absorption. The bioaccessible fractions of digested EVOOs contain water-soluble compounds dissolved in the aqueous medium and a micellar suspension formed by oil emulsified by bile salts. The different phenolic profiles of EVOOs from different varieties are probably responsible for the variations observed between oils after the SGD procedure performed on them since phenolic compounds are hydrolyzed by the intestinal enzymes, and they also undergo different structural modifications due to the conjugation process [10].

When Dinnella et al. [5] evaluated the bioaccessibility of phenolic compounds from EVOO 1–EVOO 10 by means of the bioaccessibility index (calculated as the relationship between the Ty concentration in the dialyzed solution-phase contained in the tube and the Ty equivalents expected in the dialyzed solution-phase on the basis of bioaccessibility model), such index varied from a maximum of 90% to a minimum of 37% (Table 2). The TPC and AC of EVOOs before digestion did not give significant results correlated with either the bioaccessibility index or the AC determined for the bioaccessible fraction. It is commonly accepted that differences in the AC of phenolic compounds are strongly influenced by their chemical structures. In the same way, when Borges et al. [10] assessed the bioaccessibility of the phenolic fractions of EVOO 11–EVOO 16, they observed that the effect of the digestive process on TPC varied from a 2.5-fold increase for Picual variety to a 4.1-fold increase for Hojiblanca. The highest values for the bioaccessible fraction were observed with Cornicabra (891 mg CAE/kg), Picudo (764 mg CAE/kg), Hojiblanca (689 mg CAE/kg), and Manzanilla (685 mg CAE/kg) varieties. Picual (630 mg CAE/kg) and Arbequina (613 mg CAE/kg) presented the lowest ones (Table 2). The AC also increased in digested samples—from 1.9- to 4.2-fold—compared to the oil extracts. Seiquer et al. [12] also found the same behavior when they studied the bioaccessibility of phenolic compounds from Picual EVOO 17; although the evaluation of the TPC in oil revealed 368 mg GA/kg, the bioaccessible fraction revealed 1029 mg GA/kg, which supposes an enhancement of almost three times the initial content (Table 2). For AC, the evaluation after the in-vitro SGD revealed 0.88 mmol Trolox/kg, which supposes an enhancement of almost 1.35 times the initial content.

An increase in the TPC determined in the bioaccessible fraction of the oils obtained from the Arbequina variety cultivated in different Brazilian and Spanish regions has been observed with respect to the oil extracts—from 1.5- to 6.3-fold—(Table 2). Although significant variations were found in the TPC among oils, no relationships with altitude or climatic factors were detected, contrary to those observed in the oil extracts [17].

Moreover, although antioxidant properties were strongly related to TPC in oil extracts, no relationships were found in the bioaccessible fraction after the digestive process, suggesting that after digestion, compounds other than phenolic compounds could also be responsible for antioxidant activity. On the other hand, when bioaccessibility was studied with different Spanish cultivars from different years of harvest and crop stages (EVOO 29–EVOO 36, [32]), the bioaccessible fractions showed higher values of TPC and higher AC compared with the extracts. In addition, the TPC was not affected by cultivar, and the free-radical scavenging activity (ABTS and DPPH) of the bioaccessible fractions was higher in Arbequina than in Hojiblanca, in contrast to what was shown by the oil extracts (Table 2).

From the results presented above, it can be concluded that following the in-vitro SGD, relatively high bioaccessibility values could be observed. All these results support the idea that in-vitro digestion is a crucial step that releases large amounts of phenolic and antioxidant compounds. Phenolic compounds could be transformed during digestion into different structural forms with different chemical properties, especially after the intestinal phase, since phenolic compounds are highly sensitive to alkaline conditions. The mechanisms by which glycosides may be hydrolyzed in the small intestine and other changes caused in the conjugation process could strongly affect the biological activities and AC of phenolic compounds. Some of the major olive oil phenols (HTy, Ty, and lignans) are relatively stable after gastrointestinal digestion and are able to generate derivative compounds thanks to digestion, whereas others (flavonoids and secoiridoids) are unstable [18].

The digestion process is essential to define the antioxidant properties of oils, and changes produced during digestion should be considered to predict the healthy potential of oils in-vivo. During the digestive process, antioxidants probably undergo modifications that increase their reactivity—especially due to the changes in pH, which could affect the racemization of molecules, creating enantiomers with different biological reactivity [51]. HTy and Ty presented increased recoveries during the digestive process due to the hydrolysis of secoiridoid derivatives, and they have been recognized as the most efficient free-radical scavengers and radical chain breakers, respectively [52,53]. In this line, Soler et al. [18] measured the individual phenolic compounds in oil digesta and aqueous micellar phases and observed good stability of the major compounds, especially HTy and Ty, under gastric and intestinal conditions. Taking into account that these phenolic compounds have been associated with a high level of AC, their stability may contribute to the increasing AC during digestion.

#### 3.1.3. Bioavailable Fractions

After the in-vitro SGD, some authors subjected the digested extracts to a bioavailability study with Caco-2 cells. The proportions of the total recovered phenols and their metabolites in the extracellular culture medium (apical-AP and basolateral-BL) and the cytoplasmatic contents of the cells were studied. Among all the EVOOs studied above, Picual (EVOO 17) and Arbequina (EVOO 18–EVOO 28) oils were subjected to transepithelial transport studies; the absorption of TPC and the antioxidant properties of digested oils across Caco-2 cell monolayers after 2 h of incubation were studied (Table 2 and Table 3). Seiquer et al. [12] reported that only 25% of the TPC from the digested Picual EVOOs crossed the intestinal cells. Results obtained with Arbequina EVOOs from Brazil and Spain showed that absorption of TPC ranged from 32.5 to 110%, which could be explained by two hypotheses [17]. Firstly, Folin–Ciocalteu method is not a selective assay, determining all kinds of phenolic molecules, and thus, a variety of compounds may interfere with the reactive to give apparently elevated phenolic concentrations [54]. Secondly, the metabolites present in the basal medium could have been produced intracellularly and excreted to the exterior or produced directly by secreted enzymes [18]. The average absorption of phenolic compounds was higher in Brazilian than in Spanish oils (97% vs. 67%, respectively) [17]. For Picual EVOOs, the remaining antioxidant activity levels measured by ABTS, DPPH, and FRAP in the BL compartments after incubation were only 33%, 2%, and 6%, respectively [12]. For Arbequina EVOOs recovered in the basal chambers, DPPH activity was only 30.7–52.4% of what it was before. Statistical differences were found between individual samples and between countries (51% vs. 43% for Brazilian and Spanish oils, respectively). The results suggest good bioavailability of the antioxidant properties of oils, which were maintained after digestion and absorption [17].

#### 3.1.4. Residual Fractions

The residual fraction after digestion is not considered when studying phenolic bioaccessibility. However, a large amount of phenolic compounds usually escapes absorption in the small intestine, reaching the large one. It has also been shown that unabsorbed phenolic compounds may exert beneficial local effects on the intestinal tract by interacting with the microbiota, mucosal cells, and dendritic projections in the lumen [55]. Most of these compounds can be used by gut microbes as substrates and yield others—usually low molecular weight compounds that may be absorbable through the colonic epithelium [56,57]. Saura-Calixto et al. [58] studied the bioaccessibility of TPC in a whole diet and also showed that small amounts of them (10%) were associated with the residues and remained in the food matrices after the digestion process. It has been suggested that non-absorbable phenolic compounds exert local antioxidant effects in the gastrointestinal tract.

Regarding the residual fraction from EVOOs described in this section, significant amounts of TPC, about 2.82% of the total phenols, were found by Seiquer et al. [12]; and 6.7% (DPPH) remains non-absorbable after the in-vitro digestion of EVOO. In the case of Spanish and Brazilian digested Arbequina EVOOs (EVOO 18–EVOO 28), residual fractions accounted for 17.3–47.2% of TPC and 7.0–18.5% of DPPH from the totals recovered after digestion [17]. Regarding the residual or non-soluble fraction from digested Hojiblanca and Arbequina EVOOs (EVOO 24–EVOO 31), 18–52% of the TPC remained after digestion although no DPPH activity was observed [32].

### 3.2. Assessment of Phenolic Profiles in Oils and Their Bioaccessibility by LC Methods

In order to evaluate the digestive stability of the phenolic compounds with higher precision, several advanced analytical techniques (i.e., HPLC-DAD, HPLC-MS/MS, UHPLC-HR-MS, and UHPLC-QTOF-MS) have been used to characterize the individual phenolic compounds present in the olive oil fractions obtained after in-vitro digestion.

Rubió et al. [9] characterized the bioaccessible fraction after in-vitro digestion of an olive oil extract by HPLC-MS/MS. The results showed that the different portions of bioaccessible secoiridoids were due to HTy, which showed a bioaccessible percentage of over 100% (132.2%). This could have been due to the release of HTy during the digestion of its precursors, hydroxytyrosol acetate (HTy-Ac) and the secoiridoids (HTy linked to the dialdehydic form of elenolic acid and HTy linked to elenolic acid) [27]. Moreover, the low stability of the secoiridoid derivatives during the intestinal digestion phase is mainly due to alkaline conditions [59]. Concerning flavonoid family, only luteolin (Lut) was found in the bioaccessible fraction (as in the extract) but at a very low concentration (only 14.6%) was bioaccessible.

On the other hand, three studies addressed the stability of the phenolic compounds of VOO after in-vitro digestion by using different analytical techniques [20,31,33]. Quintero-Flórez et al. [31] studied five Spanish monovarietal VOOs from Habichuelero, Sevillana, Chetoui, Picual, and Blanqueta varieties; meanwhile, Rocchetti et al. [33] evaluated five different commercial EVOOs from different geographical areas (Leccino and Frantoio from Italy, Picual from Spain, Picholine marocaine from Northern Africa, and Kalamon from Greece). More recently, Reboredo-Rodríguez et al. [20] evaluated a commercial EVOO obtained by co-crushing Galician Brava Gallega and Mansa de Figueiredo old autochthonous varieties.

A total of 26 compounds were determined by Quintero-Flórez et al. [31] by using a UHPLC-HR-MS where the quantification was performed by DAD at 280 and 360 nm, and by MS for the secoiridoid derivatives that co-eluted with other phenolic compounds with an equal response factor. Rocchetti et al. [33] determined and quantified 67 phenolic compounds by using a UHPLC-QTOF-MS. Finally, Reboredo-Rodríguez et al. [20] determined a total of 21 phenolic compounds by HPLC-DAD/FLD/MS where the quantification was performed by MS except for Lut, Apigenin (Api), and Diosmetin (Dios) (quantified by DAD (λ = 330 nm)) and for Pin and Ac-Pin (quantified by FLD (λ_exc_ 280-λ_em_ 328 nm).

**Figure 2:**
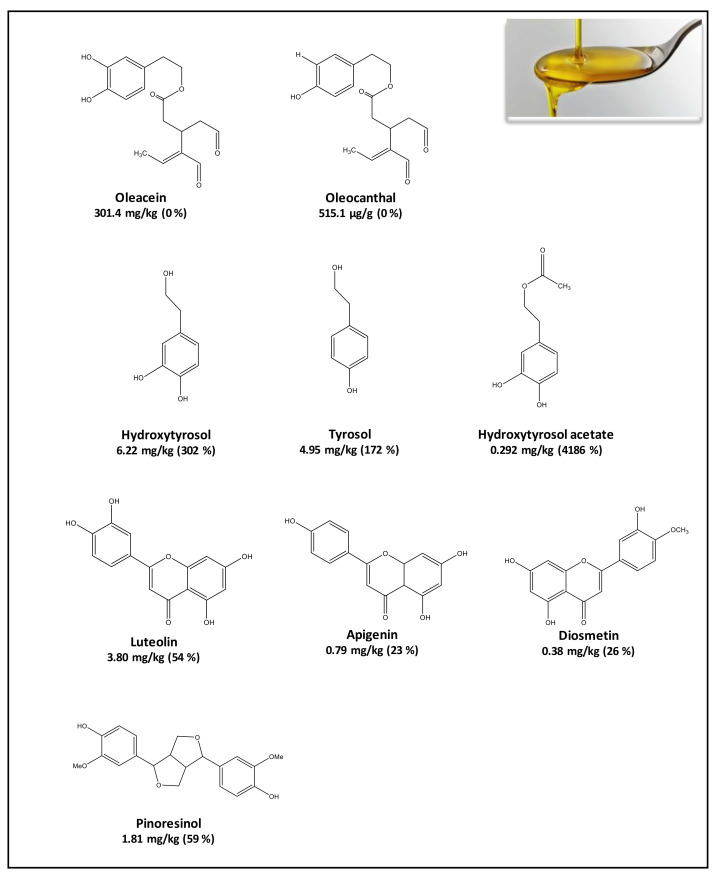
Phenolic compounds determined in EVOO (mg/kg) and their potential bioaccessibility after in-vitro digestion (%). Data taken from Reboredo-Rodríguez et al. [20].

A common behavior has been observed in these studies. Secoiridoids, which are complex phenols, including Ole and ligstroside (Lig) derivatives, are the most abundant family in the EVOO polar fraction (Figure 2). Their stability was very low when they were exposed to small-intestinal conditions [20], showing low bioaccessibility (2–10%) [20,31]. Their hydrolysis is responsible for the release of simple phenols HTy, HTy-Ac, and Ty [9,18,59], which were stable under the conditions of intestinal digestion [27]. Lignans and flavonoids, which remain manly in the fraction directed to the colon, could interact with the intestinal microbiota, which facilitates their degradation and transformation into substances with low molecular weight and potentially absorbable structures in the colon [56,60]. In general, phenolic acids were scarcely bioaccessible or even disappeared because they were unstable in the intestinal conditions [20].

## 4. Table Olives

Table olives are a typical food of the MedDiet and are considered antioxidant-rich foods [28]. Antioxidant, anti-inflammatory, and antitumoral properties of table olives are due to the presence of phenolic compounds, which represent 1–3% of the mean composition of the fruits [4,61]. Unprocessed table olives are rich in Ole and Lig, which are both responsible for their bitter taste.

Olives must be processed to be consumed as table olives. Table olive processing involves the removal of the bitter taste by hydrolysis of Ole with alkali (1.8–2.5% sodium hydroxide aqueous solution), followed by washing to remove the excess alkali, and/or olives can be soaked with brine (5–10% sodium chloride). In most cases, the subsequent fermentation driven by yeasts and acid lactic bacteria imparts to the fruits a well-defined sensory profile, and the decrease in pH as a result of the production of lactic acid prevents the growth of pathogenic microorganisms [28,29,30]. According to the major companies, about 80% of the world’s production is covered by three commercial processing methods: treated green olives (or Spanish-style green olives); olives darkened by oxidation (ripe olives) (Californian style); natural (mainly black) olives (Greek style) [62]. Technological factors can considerably affect the qualitative and quantitative phenolic composition [63]. The main phenolic compounds of debittered table olives correspond to HTy and Ty and comselogoside isomers (the major oleosides in olive fruit) [61]. In addition, verbascoside (VB, as the main hydroxycinnamic derivative) and Lut and Api (flavonoid derivatives) are also present [29]. The phenolic content of table olives is about five times higher than in olive oil [29].

**Figure 3:**
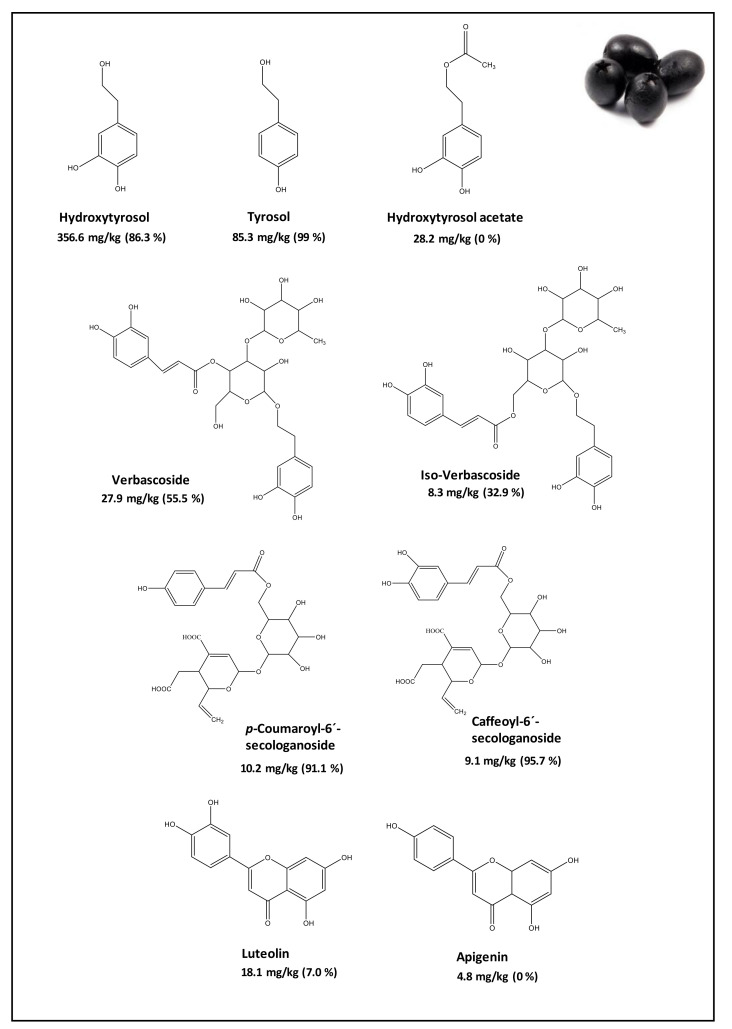
Phenolic compounds determined in olives (mg/kg) and their potential bioaccessibility after in-vitro digestion (%). Data taken from D’Antuono et al. [29].

To date, there have been few research papers focused on the evaluation of their bioaccessibility using an in-vitro SGD model. D’Antuono et al. [29] assessed the bioaccessibility of the naturally fermented (Greek style) Bella di Cerignola (BdC) table olives. Initially, the phenolic profile of BdC debittered table olives was established by HPLC-DAD and HPLC-MS/MS. Phenolic compounds responsible for the TPC (548.5 mg/kg of fresh weight—FW) were, in particular, HTy (356.6 mg/kg), Ty (85.3 mg/kg), HTy-Ac (28.2 mg/kg), VB (27.9 mg/kg), Lut (18.1 mg/kg), isoverbascoside (isoVB, 8.3 mg/kg), caffeoyl-6′-secologanoside (SEC, 9.1 mg/kg), comselogoside (COM, 10.2 mg/kg), and Api (4.8 mg/kg). The highest relative abundances of HTy (65%) and Ty were associated with hydrolysis of Ole and Lig during the table olive processing (as can be seen in Figure 3). According to these authors, after the SGD, the same phenolic compounds remained, but their contents changed. The percentage of bioaccessibility for each compound was calculated by dividing the phenolic concentration in the aqueous small intestinal digesta by the initial phenolic concentration in the undigested flesh of table olives. Those phenolic compounds with higher solubility in the aqueous medium, such as digesta fluids, were released in higher amounts (>86%, see Figure 3). The high HTy bioaccessibility could also have been due to the release from its precursors (VB and HTy-Ac) during the digestion. In fact, the bioaccessibility of VB (55.5%) and isoVB (32.9%) resulted unstable; Api was not detected in the digesta, and Lut showed a very low bioaccessibility of about 7%.

The same authors assessed the effects of select microbial starters (for fermentation) on the phenolic compositions of Italian table olives of different cultivars (BdC; Termite di Bitetto—TDB; and Cellina di Nardò—CEL) and their antioxidant activities and bioaccessibility [28]. Commercial samples obtained from fermentation driven by autochthonous yeasts and bacteria were used as controls. For BdC table olives processed with starters (1194 mg/kg fresh weight—FW), the phenolic total doubled compared with a commercial sample (609.5 mg/kg FW). For TDB (349.6 mg/kg FW) and CEL (2238.7 mg/kg FW), phenolic compounds remained almost unchanged. However, CEL table olives processed with starters presented the highest AC (21.7 mg Trolox eq/g FW) compared to the other cultivars. In general, phenolic compounds were highly bioaccessible (>60%) in all varieties of olives studied. In commercial BdC samples, HTy and Ty showed good bioaccessibility (63 and 78% without starters; 52 and 60% with starters). In BdC with starters, SEC, COM, and caffeic acid (CA) derivatives had higher bioaccessibility than in commercial BdC samples, whereas VB and isoVB had worse bioaccessibility in the former. The behavior was similar for TDB and CEL digesta samples. Lut and Api were poorly bioaccessible (<5%), and they were not detected in the chime of the starters of the fermented olives [28].

In agreement with other authors, Ole and COM isomers were the main phenolic compounds, followed by VB and elenolic acid and its derivatives, when other varieties of olives were studied [4,30]. Fernández-Poyatos et al. [30] quantified by HPLC-MS/MS individual phenolic compounds in fermented Cornezuelo table olives. Total individual phenolic content (TIPC) was about 12,800 mg/kg of lyophilized sample; meanwhile, TIPC decreased after the simulated digestion by approximately 50%, down to 5800 mg/kg in the soluble fraction and 450 mg/kg in the residual fraction. This decrease after SGD was greater than what was observed by the authors cited above. In more detail, 25% of Ole and 20% of COM remained stable in the soluble fraction.

**Figure 4:**
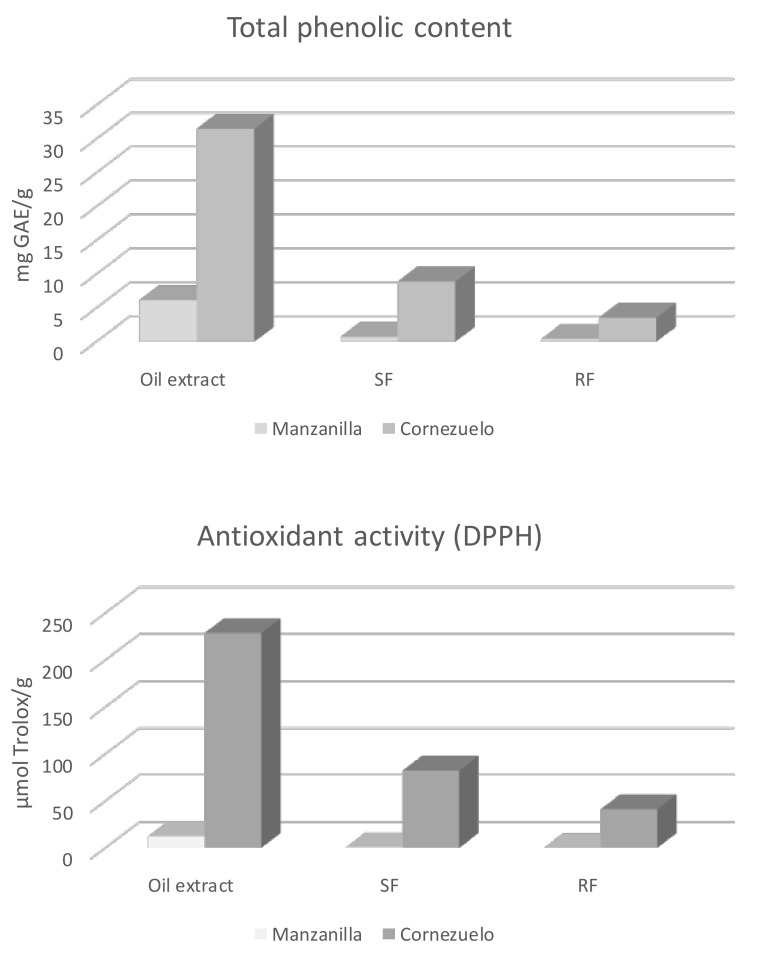
Total phenolic contents (TPC) and antioxidant capacities (AC) of Manzanilla and Cornezuelo table olive extracts (before SGD), SF (soluble fractions after SGF; liquid), and RF (residual fractions after SGF; solid). Data taken from Fernández-Poyatos et al. [30].

Although HPLC-MS/MS allows one to assess individual phenolic bioaccessibility, conventional spectrophotometric assays to determine TPC and AC are still widely used by many authors to compare their results with others. The results obtained by spectrophotometric methods are in line with those obtained by HPLC-MS/MS. Decreases in the amounts of phenolic compounds and AC of digested samples were observed compared to non-digested table olives, as is depicted in Figure 4.

After SGD, there were losses of approximately 72 and 89% of TPC in the Cornezuelo and Manzanilla soluble fractions, respectively; however, significant AC was observed in both soluble fractions—around 36% of the initial antioxidant values for Cornezuelo oils [30]. The residual fraction had approximately 10% of the TPC in comparison to the non-digested samples, which could be metabolized by the colon microbiota [4,30].

## 5. Residues from Olive Oil Production

Olive oil byproducts have been valorized by the olive oil industry to increase sustainability and build the circular bioeconomy [40,41]. It is good to extract and recover phenolic compounds from olive industry byproducts due to their being abundant, largely unexploited, low-cost sources of powerful antioxidants that can also be used in food and pharmaceutical industries.

### 5.1. Olive Leaves

Olive leaves are considered an agricultural residue of olive tree pruning [64] and a byproduct in the olive industry (olive leaves represent 10% of the total weight of the olives arriving at mills) [35,37,39,65]. Olive leaves are considered an interesting source of bioactive compounds; great interest has been mainly shown by cosmetic and pharmaceutical industries [36,66]. Their high levels of phenolic compounds are of particular note due to their health properties [36].

Secoiridoids are the main chemical group of phenolic compounds in olive leaf extracts. Ole is the main compound, followed by HTy and VB, apigenin-7-glucoside (Api-7-glucoside), luteolin-7-glucoside (Lut-7-glucoside), and Ty. These compounds possess antioxidant, antimicrobial, anti-inflammatory, cardioprotective, antihypertensive, hypoglycemic, and hypocholesterolemic properties [38,67]. Furthermore, Ole has hypocholesterolemic and hypoglycemic properties [49,67,68,69].

To use olive leaves to benefit human health, drying and extraction operations to obtain their extracts must ensure not only the stability necessary for later applications but also the maintenance of antioxidant potential [35]. In this sense, Ahmad-Qasem et al. [35] assessed hot air drying at 70°C (HAD-70) and 120°C (HAD-120) and freeze-drying using conventional freezing at −28°C (FD) as drying processes; meanwhile, extraction was performed with ethanol/water (80:20, *v/v*) using conventional and ultrasound-assisted (US) extractions. Concerning drying process, HAD-120 and FD extracts showed the highest TPC and AC (66 mg GAE/g dw and 102.2 mg Trolox/g dw) and the lowest TPC and AC (33 mg GAE/g dw and 75 mg Trolox/g dw), respectively. Although HAD-120 followed by US promoted the formation of free radicals, individual contents of the main phenolic compounds were higher for HAD-120 without US extraction: Ole (65.0 mg/g dw), VB (18.5 mg/g dw), and Lut-7-glucoside (11.0 mg/g dw). Once the processing conditions were optimized, the stability of the bioactive phenolic compounds was assessed under SGD. A significant decrease in desirable compounds (31.4% in TPC) in HAD-120 extracts was observed within the first hour of gastric digestion as a consequence of the degradation of Ole, VB, and Lut-7-glucoside produced by pH changes and enzymatic activity. Pancreatic enzyme activity and the alkaline pH of intestinal digestion contributed to the total disappearance of VB and Ole, but meanwhile, Lut-7-glucoside was fairly resistant to digestion and therefore should be considered an interesting phenol for further absorption experiments.

It is important to note that the use of organic solvents, such as methanol and hexane, as extraction solvents has been rejected owing to their toxicity [36]. In addition, the quantity of phenolic compounds present in aqueous extracts is considerably higher than that found in hydromethanolic extracts [38,70]. Following a strategy proposed by several papers, olive leaves can be partially dried in a conventional oven, and bioactive compounds can generally be extracted from leaves with ultrapure water at 60–80 °C [35,36,38].

**Figure 5:**
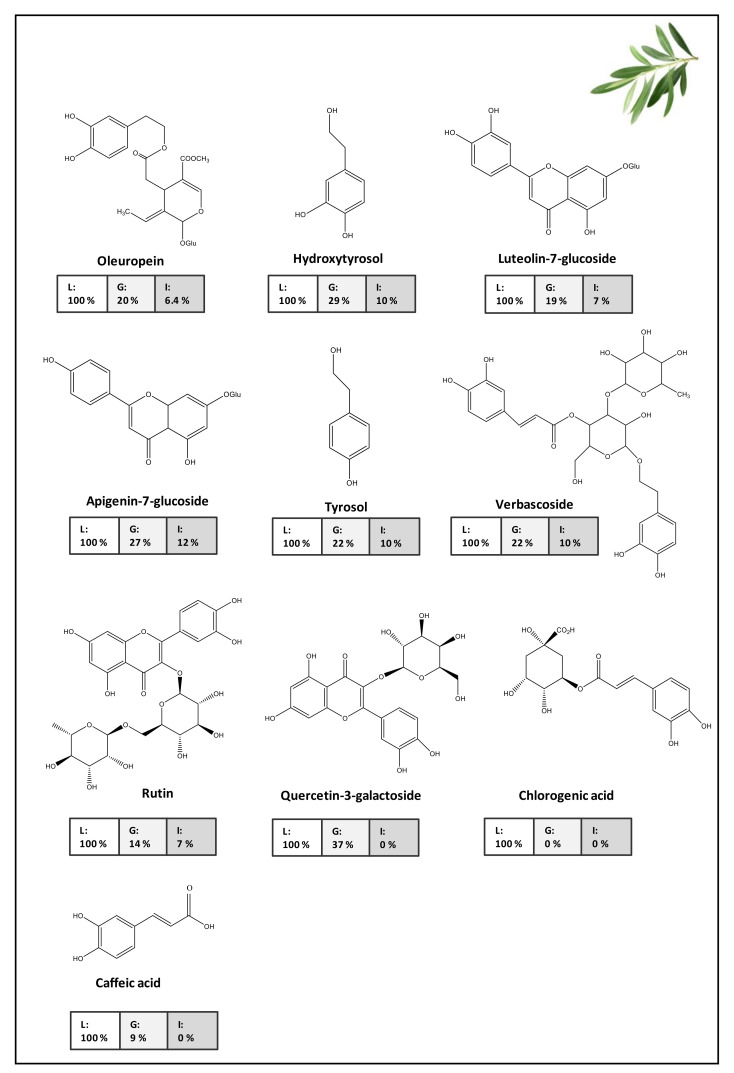
Phenolic compounds determined in leaves. Variation (%) of the phenolic compounds after gastric (G) and intestinal (I) digestion with respect to the initial content in leaves (L). Data taken from Martín-Vertedor et al. [36].

As can be seen in Figure 5, the olive leaf aqueous extract possessed high contents of Ole (more than 50%, 22,541.8 mg/kg), HTy (10,924.7 mg/kg), Lut-7-glucoside (3741.9 mg/kg), Api-7-glucoside (2147.1 mg/kg), Ty (1681.2 mg/kg), and VB (914.4 mg/kg), which represented more than 90% of total phenolic compounds. CA (201.5 mg/kg), chlorogenic acid (30.72 mg/kg), and quercetin-3-*O*-galactoside (30.57 mg/kg) were the minor compounds and represented 1%. The AC of the olive leaf extract was equivalent to about 15.6 g Trolox/kg extract, and when it was compared to VOO, it was found to be 15-fold higher [36]. Therefore, based on these results, phenolic compounds from olive leaves could be a good strategy with which to increase the phenolic content of VOO and therefore, to develop functional VOOs [36,42,71]. After the SGD, phenolic content was reduced with respect to the olive leaf extract (as it shown in Figure 5). After the enzymatic activity in the oral phase, a significant (*p* < 0.05) decrease (around 30%) in the total phenolic content was registered; HTy, Ole, and Lut-7-glucoside suffered decreases of 23, 22, and 17%, respectively, and other minor compounds were not detected after oral phase simulation [38]. After gastric digestion, the initial quantities of phenolic compounds were reduced by 35–70% [36,38]; HTy, Ty, Ole, Api-7-glucoside, and VB decreased 51, 57, 66, 69, and 64%, respectively, compared to the fresh extract and 36, 54, 56, 67, and 62% compared to the oral step. Finally, after the small intestine phase, the phenolic content decreased by around 90% compared to the fresh extract. The most important decrease was suffered by Ole (Figure 5) [36,38]. Although only 10% of the phenolic content was available to be absorbed in the intestinal tract, antioxidant, antimicrobial, and antitumor activities against human histiocytic leukemia U937 cells remained substantial enough to consider the aqueous olive leaf extract for therapeutic use [36,38]. Phenolic compounds present in aqueous olive leaf extracts are unprotected from adverse environmental conditions and/or gastrointestinal conditions. In order to increase and protect their bioactivities, bioaccessibility, and bioavailability in the gut, different biosorption and/or encapsulation strategies were recently proposed [37,72].

The biosorption of the phenolic compounds of olive leaf infusions into *Saccharomyces cerevisiae* was proposed by Jilani et al. [37] as a promising delivery system and an effective means to protect the bioactivity during digestion. The biosorption on *S. cerevisiae* allowed the recovery of 25.17 mg/g–49.40% of the phenolic compounds from var. Chemlali olive leaf infusions. This result confirmed the ability of dried yeast cell walls to adsorb phenolic compounds; phenolic compounds with higher molecular weights tended to more strongly bind to yeast cell walls. The optimum adsorbent amount was 10 g yeast/L, and equilibrium was reached after almost three hours. Both infusions and suspensions before and after biosorption were analyzed by spectrometric methods. TPC in the initial olive leaf extract before biosorption (BB-BD) was 622.96 mg GAE/L, and it was reduced to 538.18 mg/L in the bioaccessible extract when SGD was applied (BB-AD). TPC in the initial olive leaf extract after biosorption (AB-BD) was 409.68 mg GAE/L, but it was increased to 595.40 mg/L in the bioaccessible extract when SGD was applied (AB-AD). The binding of olive leaves’ phenolic compounds to the yeast allowed bioaccessibility for over 45% of them. With respect to the AC determined by TEAC, olive leaf extract BB-BD presented 2.44 mM of Trolox equivalents, which was increased to 3.64 mM TE in the bioaccessible extract (BB-AD). For another olive leaf extract (AB-BD), the result was 1.83 mg GAE/L, but this was increased to 5.04 mg/L (AB-AD) in the bioaccessible extract. In light of the TEAC results, the binding of olive leaf phenolic compounds to the yeast clearly allowed bioaccessibility of over 1000%. In any case, at the end of SGD, all olive leaf extracts after biosorption had higher release rates of phenolic compounds than before biosorption. All olive leaf extracts were analyzed by TOF-HPLC-MS-MS, paying attention to Ole and HTy. The BB-AD extract presented bioaccessible portions of 68.40 and 3.07% for HTy and Ole, respectively. The AB-AD extract presented bioaccessible portions of 118.57 and 9.38% for HTy and Ole, respectively. In conclusion, large phenolic compounds could have been degraded into monomer units before being absorbed through the tract, and biosorption seems to be a useful tool to protect both phenolic compounds from SGD conditions.

Encapsulation of Ole with encapsulant agents, such as sodium alginate [73], maltodextrin (MD, [72]), or inulin (IN, [72]), is another viable technology for protecting these compounds from adverse conditions of SGD. According to these authors, Ole was spray-dried with MD or IN to evaluate the bioaccessibility and bioavailability after in-vitro SGD with respect to the non-encapsulated sample. The encapsulation efficiency was estimated by determining total and surface Ole contents. The efficiency was higher in Ole-MD (92.2%) than in Ole-IN (81.6 %) [72]. Under the adverse conditions of gastric digestion, an Ole decrease was observed in Ole-MD microparticles as a consequence of the water-solubility of MD, which favored Ole release and its exposition. As a consequence, the behavior of Ole in Ole-MD was similar to an Ole solution. Ole aglycone and HTy were formed as a result of Ole degradation. In the case of Ole-IN, hydrolysis during gastric digestion was lower. However, the Ole content declined more sharply in Ole-IN than in Ole-MD particles. The bioaccessibility of encapsulated samples was higher (15% in Ole-MD and 12% in Ole-IN) than that of non-encapsulated Ole samples (1.5%). Therefore, these authors concluded that encapsulation of Ole leads to higher Ole contents at the end of digestion, suggesting a protective role of the polysaccharides through the formation of non-covalent polysaccharide–Ole complexes. Nevertheless, further research is needed to be implemented in biosorption and encapsulation in the food industry.

Olive leaf extract has been recently accepted by the EFSA as a safe product that can be used as a functional ingredient for food fortification and/or as a food additive. In fact, the complexity of some food matrices could have been phenolic compounds, protective against the conditions of digestion. In this sense, Cedola et al. [39] developed a novel food, the “Taralli”, enriched in phenolic compounds. During the processing of this novel food, they substituted the white wine (a traditional ingredient) with an olive leaf extract. The olive leaf extract was obtained after drying olive leaves (Coratina cultivar) and extracting with ethanol/water (50:50, *v/v*). The acceptability of this novel food was initially verified by sensory analysis. Values of TPC, flavonoid contents, and AC before and after cooking were higher when white wine was substituted. These authors studied in more detail the degradation kinetics of Ole, HTy, and VB, the main phenolic compounds, during the three different digestion phases (oral, gastric, and intestinal). Cedola et al. [39] did not observe a significant decrease (*p* < 0.05) in the phenolic compounds during the oral phase, whereas the contents of target phenolic compounds were negligible after the gastrointestinal steps, and consequently, they were not bioaccessible.

### 5.2. Olive Byproducts from Olive Oil Production

Different methods are used to extract oil from olives, and these processes create large volumes of liquid and solid waste. Only 20% of olive fruit results in VOO; the remaining 80% is wasted as olive mill waste water (OMWW) and olive pomace (OP). While both OMWW and OP have been generally regarded as waste with negative environmental impacts, innovative projects are focused on reducing the environmental impacts of olive oil production by valorizing these phenolic compounds for the food and pharmaceutical industries. Phenolic compounds identified in OMWW samples include HTy, Ty, CA, *p*-coumaric acid, catechol, 4-methylcatechol, *p*-hydroxybenzoic acid, vanillic acid, syringic acid, gallic acid, catechin, Api, kaempferol, Lut, quercetin, cyanidin, peonidin, nüzhenide, Lig, VB, and some polymeric compounds (tannins and catecholmelanins) [40,74,75,76]. Phenolic compounds identified in OP samples include HTy-glucoside, HTy, Ty-glucoside, Ty, CA, VB, *p*-coumaric acid, and SEC [41]. Several in-vitro and in-vivo studies have shown that phenolic compounds from OMWWs and OP exert potent biological activities, including prevention of passive, smoke-induced oxidative stress; reduction of thromboxane B2 production by whole blood; amelioration of symptoms of inflammatory diseases, such as osteoarthritis; and reduced oxidative damage on human colon carcinoma cells (HT29) [40,75,77,78,79]. As in the previous olive matrix, the impact of SGD on phenolic compounds can be evaluated to guarantee their bioaccessibility and their benefits on health.

#### 5.2.1. Olive Mill Waste Water (OMWW) 

The production of active phenolic antioxidants from OMWW constitutes a viable alternative for transforming this agroindustrial waste stream into a useful and relevant ingredient suitable for applications in dietary supplements and/or functional foods [40,75].

While HTy from OMWW is the most studied biocompound, Cardinali et al. [79] focused on VBs, including their bioaccessibility and intestinal/uptake, using an in-vitro digestion/Caco-2 model. Initially, extraction of OMWW required the use of membranes with different porosities (micro—above 5000 Da; ultra—from 5000 to 200 Da; and nano—below 200 Da) capable of obtaining three permeated fractions. The ultra fraction was purified by gel filtration low-pressure chromatography on Sephadex LH-20 and eluted with 30% ethanol pH 5.3. Analysis of the purified ultra fraction by HPLC-DAD-ESI-MS confirmed the removal of HTy and Ty. IsoVB, followed by VB, were the main compounds. After SGD, VB’s bioaccessibility was higher (35.5%) than that of isoVB (9.2%), which was more susceptible to SGB conditions, and thereby, there was less available for absorption [79]. Both compounds were also absorbable by Caco-2 cells, which could explain their antioxidant effects on phospholipid membranes and their ability to modulate plasma’s antioxidant measures in in-vivo assays [40,80,81].

#### 5.2.2. Olive Cake (OP) 

OP (or olive cake) provides the same olive oil profile but in higher concentrations [82]. The development of OP-based ingredients has been recently studied to add value to OP and reduce its environmental impact [41]. To achieve this objective, Ribeiro et al. [41] centrifuged OP, and the resulting liquid fraction was freeze-dried with 2% mannitol. The powder obtained was denominated liquid enriched olive pomace powder (LOPP). According to these authors, LOPP has great potential as an ingredient for functional foods due to its richness in sugars and organic acids (glucose, fructose, mannitol, and formic acid), minerals (phosphorus, magnesium, calcium, and potassium), and phenolic compounds (HTy-glucoside, Ty-glucoside, HTy, Ty, and *p*-coumaric acid) and its antioxidant and antimicrobial properties [41]. After the SGD conditions, a negative effect was observed—a sharp decline in the TPC corresponding to the oral phase, from about 3000 to 180 mg GAE/g dw. Continuously, a slight increase in the stomach was observed, and the acidic pH’s protection may have caused the increases in HTy, *p*-coumaric, and CA; but decreases were produced in the intestine by the mild alkaline conditions. Just like other publications, the results obtained by HPLC-ESI-UHR-QqTOF-MS were in line with TPC results. Elenolic acid, HTy, Ty, and glucose were formed by hydrolysis of Ole after SGD. HTy and Ty were the most bioaccessible compounds with a bioaccessibility index of 82.10 and 2.59%, respectively. The low stability of phenolic compounds after SGD was reflected in the low value of AC. A good correlation of AC with TPC was registered, which confirms that phenolic compounds are the main contributors to the antioxidant properties of LOPP. On the contrary, the high AC of LOPP in the colon could cause a decrease in local oxidative stress and an improvement in microbiota composition, improving gut permeability and boosting immunity/anti-inflammatory mechanisms [41].

## 6. Conclusions

The different varieties of olive fruits are responsible for the different phenolic profiles of olives and VOOs. Regarding the in-vitro digestion process, relatively high bioaccessibility values were observed following both gastric and intestinal phases. All these results supported that in-vitro digestion is a crucial step that releases large amounts of phenolic and antioxidant compounds. HTy and Ty presented increased recovery during the digestive process due to the hydrolysis of secoiridoid derivatives, and they have been recognized as the most efficient antioxidants. The absorption of phenolic compounds across Caco-2 cell monolayers and the antioxidant properties of digested oils showed low proportions of phenolic absorption despite the high bioaccessibility previously detected. Residual fractions deserve to be considered regarding the total antioxidant power of a digested oil because unabsorbed phenolic compounds may exert beneficial local effects in the intestinal tract by interacting with microbiota, mucosal cells, and dendritic projections in the lumen. Nowadays, the interest in extracting and recovering phenolic compounds from olive industry byproducts is due to them being abundant, largely unexploited, low-cost resources of powerful antioxidants, which could be used in the cosmetic and pharmaceutical industries.

## Figures and Tables

**Table 1:** Experimental conditions of simulated gastrointestinal digestion applied to different olive matrices for the assessment of bioaccessibility.

	Oral Digestion	Gastric Digestion	Intestinal Digestion	References
**Phenolic Standards**				
Hydroxytyrosol, hydroxytyrosol acetate, and alkyl hydroxytyrosyl ethers	not performed	- Phenolic standards (10 mL), ranging from 177 to 254 µM. - pH = 2 by adding HCl 35%, *v/v.* - addition of porcine pepsin solution (4 mg, 13,040 units). - incubation at 37 °C for 2 h in a shaking water bath.	- addition of 2 mL of pancreatin (4 mg/mL) from porcine pancreas and porcine bile salts extract (12 mg/mL). - pH = 7.5 by adding NaOH 6 M. - incubation at 37 °C for 2 h in a shaking water bath. - inactivation of enzymes by adding HCl (35%, *v/v*).	[27]
**Olive Fruits**				
*O. europaea* var. Bella di Cerignola, Termite di Bitetto and Cellina di Nardò	- Olives depitted (6 g). - artificial saliva (6 mL containing 0.2 mg α-amylase/g olives, 0.4 mg mucin/g olives, 0.3 mg uric acid/g olives, and 3 mg urea/g olives). - incubation at 37 °C and rotated head-over-heels (55 rpm) for 10 min.	- addition of porcine pepsin solution (2 mL, 6.3 mg/g olives in HCl 0.1 M). - pH = 3.0 by adding HCl 1.0 M. - incubation at 37 °C for 1 h in head-over-heels (55 rpm). - addition of 0.9% saline solution.	- pH = 6.5 by adding NaHCO_3_ 1.0 M. - addition of intestinal enzymes (2.0 mL containing 10 mg pancreatin/g olives, 5 mg lipase/g olives in NaHCO_3_ 0.1 M), and porcine bile salts (3 mL, 12 mg/g olives in NaHCO_3_ 0.1 M). - addition of 0.9% saline solution. - incubation at 37 °C for 2 h in head-over-heels (55 rpm). - acidification with 2% aqueous acetic acid (1:1).	[28]
*O. europaea* var. Bella di Cerignola	- Flesh olives (6 g). - artificial saliva (6 mL containing 10.6 g α-amylase/g olives, 5% mucin (*w/v*), 3% uric acid (*w/v*), and 40% urea (*w/v*)). - incubation at 37 °C and rotated head over heels (55 rpm) for 10 min.	- addition of porcine pepsin solution (2 mL, 20 mg/mL in HCl 0.1 M). - pH = 3.0 by adding HCl 0.1 M. - incubation at 37 °C for 1 h in a covered shaking water bath (85 rpm). - addition of 0.9% saline solution.	- pH = 6.5 by adding NaHCO_3_ 1.0 M. - addition of intestinal enzymes (2.0 mL containing 30 mg/mL pancreatin, 15 mg/mL lipase in NaHCO_3_ 0.1 M), and porcine bile salts (3 mL, 120 mg/mL bile extract in NaHCO_3_ 0.1 M). - addition of 0.9% saline solution. - incubation at 37 °C for 2 h in a shaking water bath. - acidification with 2% aqueous acetic acid (1:1).	[29]
*O. europaea* var. Cornezuelo	- Lyophilized olives (2 g). - artificial saliva (4 mL containing 2.12 g α-amylase/mL, 1 mg mucin/mL, and 0.4 mg urea/mL). - incubation at 37 °C and agitation for 5 min.	- addition of gastric juice (10 mL containing 5 mg pepsin /mL, 6 mg mucin /mL, and 0.18 mg urea/mL). - pH = 1.30. - incubation at 37 °C for 2 h in a shaking water bath.	- pH = 8.1 by adding NaHCO_3_ 1.0 M. - addition of duodenal juice (10 mL containing 18.04 mg pancreatin/mL, 3 mg lipase/mL, and 0.2 mg urea/mL) and bile juice (4 mL containing 24.02 mg bile salts/mL and 0.52 mg urea/mL). - incubation at 37 °C for 2 h in a shaking water bath. - samples were frozen at −80 °C to stop reaction.	[30]
*O. europaea* var. Manzanilla	- Table olives (10 g). - artificial saliva (containing α-amylase (1500 U/mL)). - incubation at 37 °C and agitation for 10 min.	- addition of gastric juice containing pepsin (2500 U/mL). - pH = 3.0 by adding HCl 6 M. - incubation at 37 °C for 1 h in a shaking water bath.	- addition of duodenal juice (pancreatin (800 U/mL) and bile salts (160 mM)). - pH = 7.0 by adding NaOH, 1 M. - incubation at 37 °C for 2 h in a shaking water bath. - the liquid soluble fraction was centrifuged at 10,000 rpm for 10 min at 4 °C for later analysis.	[4]
**Olive Oils**				
*O. europaea* var. Oliarola del Bradano, Maiatica, Coratina	not performed	- Olive oil samples (10 g) were diluted with distilled water (1:8, *w/v*). - pH = 2.0 by adding HCl 6 N. - addition of pepsin solution (3 mL of a pepsin solution (160 mg/mL) from pig gastric mucosa in HCl 0.1 N). - addition of distilled water to reach a final volume of 100 mL. - incubation at 37 °C for 2 h in a shaking water bath.	- pH = 5 by adding NaHCO_3_ 0.9 N. - addition of pancreatin/bile solution (24.5 mL containing pancreatin (4 mg/mL) and bile (25 mg/mL) in NaHCO_3_ 0.1 M). - pH = 7.0 by adding NaHCO_3_ 0.1 M. - The mixture was stirred at 37 °C for 2 h.	[5]
*O. europaea* var. Arbequina, Cornicabra, Manzanilla, Hojiblanca, Picual, Picudo	not performed	- Olive oil samples (4 g) mixed with Milli-Q water (1:10, *w/v*). - pH = 2.0 by adding HCl 6 N. - addition of 0.313 mL of pepsin/0.1 N HCl (160 mg pepsin per mL). - incubation (110 oscillations per min) at 37 °C for 2 h in a shaking water bath.	- pH = 6 by adding NaHCO_3_ 1 M. - addition of pancreatin/bile salts solution (2.5 mL, containing pancreatin (4 mg/mL) and bile salts (2.5 mg/mL) in NaHCO_3_ 0.1 M). - incubation (110 oscillations per min) at 37 °C for 2 h in a shaking water bath. - inactivation of enzymes by heat treatment (4 min, 100 °C).	[10]
*O. europaea* var. Picual	not performed	- Olive oil samples (1 g) mixed with bi-distilled deionized water (9 mL). - pH = 2.0 by adding HCl 1 N. - addition of 0.05 g of pepsin/g sample (0.8 g of pepsin dissolved in 5 mL of HCl 0.1 M). - incubation (110 oscillations per min) at 37 °C for 2 h in a shaking water bath.	- pH = 7.5 by adding NaHCO_3_ 1 M. - addition of pancreatin and bile salts mixture (2.5 mL, containing 0.1 g of pancreatin and 62.5 mg of bile salts dissolved in 25 mL of NaHCO_3_ 0.1 M). - incubation (110 oscillations per min) at 37 °C for 2 h in a shaking water bath. - inactivation of enzymes by heat treatment (4 min, 100 °C).	[12]
*O. europaea* var. Arbequina	not performed	- Olive oil samples (4 g) mixed with Milli-Q water (1:10, *w/v*). - pH = 2.0 by adding HCl 6 N. - addition of 0.313 mL of pepsin/0.1 N HCl (160 mg pepsin per mL). - incubation (110 oscillations per min) at 37 °C for 2 h in a shaking water bath.	- pH = 6 by adding NaHCO_3_ 1 M. - addition of pancreatin/bile salts solution (2.5 mL, containing pancreatin (4 mg/mL) and bile salts (2.5 mg/mL) in NaHCO_3_ 0.1 M). - incubation (110 oscillations per min) at 37 °C for 2 h in a shaking water bath. - inactivation of enzymes by heat treatment (4 min, 100 °C).	[17]
*O. europaea* var. Picual, Blanqueta, Sevillana, Habichuelero and Chetoui	- Olive oil samples (5 g). - addition of artificial saliva (6 mL). - incubation at 37 ± 2 °C and rotated head over heels (55 rpm) for 5 min.	- addition of gastric juice (12 mL). - pH = 2–3. - incubation at 37 ± 2 °C and rotated head over heels (55 rpm) for 2 h.	- addition of duodenal juice (12 mL), bile (6 mL), and NaHCO_3_ solution (1 M, 2 mL). - pH = 6.5–7. - incubation at 37 ± 2 °C and rotated head over heels (55 rpm) for 2 h.	[31]
*O. europaea* var. Arbequina, Hojiblanca	not performed	- Olive oil samples (4 g) mixed with Milli-Q water (1:10, *w/v*). - pH = 2.0 by adding HCl 6 N. - addition of 0.313 mL of pepsin/0.1 N HCl (160 mg pepsin per mL). - incubation (110 oscillations per min) at 37 °C for 2 h in a shaking water bath.	- pH = 6 by adding NaHCO_3_ 1 M. - addition of pancreatin/bile salts solution (2.5 mL, containing pancreatin (4 mg/mL) and bile salts (2.5 mg/mL) in NaHCO_3_ 0.1 M). - incubation (110 oscillations per min) at 37 °C for 2 h in a shaking water bath. - inactivation of enzymes by heat treatment (4 min, 100 °C).	[32]
*O. europaea* var. Leccino Frantoio, Picual, Picholine marocaine, Kalamon	- Olive oil samples (scaled up to 500 µL of liquid sample). - SSF at pH 7.0 with salivary α-amylase (75 U/mL). - Incubation at 37 °C for 2 min.	- mixture with the SGF (ratio 1:1) at pH 3.0 (HCl 1 M) containing porcine pepsin (2000 U/mL). - incubation at 37 °C for 2 h.	- mixture with the SIF (ratio 1:1) at pH 7.0 (NaOH 1 M) containing pancreatin (100 U/mL) and bile salts (10 mM). - incubation at 37 °C for 2 h.	[33]
*O. europaea* var. Manzanilla, Picual	- Olive oil samples (5 g). - addition of SSF (5 mL) with α-amylase and CaCl_2_ 0.3 M (25 µL). - Incubation at 37 °C for 2 min.	- addition of SGF (10 mL) with pepsin and CaCl_2_ 0.3 M (5 µL). - pH = 3.0 by adding HCl 1 N. - Incubation at 37 °C for 2 h.	- addition of SIF (20 mL) with pancreatin, bile salts, and CaCl_2_ 0.3 M (40 µL). - pH = 7.0 by adding NaOH 1 N. - incubation at 37 °C for 2 h.	[34]
*O. europaea* var. Brava Gallega, Mansa de Figueiredo	- EVOO samples (5 g). - addition of SSF (5 mL). - Incubation at 37 °C for 2 min (pH = 7).	- addition of SGF (10 mL) containing pepsin (2000 U/mL) and gastric lipase (60 U/mL). - pH = 3. - incubation at 37 °C for 2 h.	- addition of SIF (20 mL) containing bile salts (10 mM) and pancreatin (100 U/mL). - pH = 7. - incubation at 37 °C for 2 h.	[20]
**Olive Leaves**				
*O. europaea* var. Serrana	- Olive leaf extracts (10 g) diluted with distilled water (1:8 *w/v*). - pH = 2.0 by adding HCl 6N. - stirring for 15 min.	- pH = 2 by adding HCl 6N. - addition of pepsin (3 mL, 160 mg/mL, 3.8 units/mg protein) from pig gastric mucosa in HCl 0.1 N). - incubation at 37 °C for 2 h.	- pH = 5 by adding NaHCO_3_ 0.9 M - addition of pancreatine-bile solution (22.54 mL, containing pancreatin (4 mg/mL) and bile (25 mg/mL) in NaHCO_3_ 0.1 M). - pH = 7.0 by adding NaHCO_3_ 0.1 M. - incubation at 37 °C for 2 h in a shaking water bath.	[35]
*O. europaea* var. Arbequina	- Olive leaf extracts (1 g). - addition of oral fluid which contained 1 mL of human saliva/g extract. - Incubation at 37 °C for 20 s.	- addition of SGF (3.6 mL, containing 0.2 g pepsin and 0.125 g NaCl in deionized water). - pH = 2.2. - incubation at 37 °C for 20 min.	- pH = 6.5 by adding NaOH. - addition of SIF (3.6 mL, containing pancreatin (20 mg), lipase (5 mg), cholic acid (10 mM), and deoxycholic acid (10 mM) in PBS buffer 0.1 M). - incubation at 37 °C for 20 min in a shaking water bath.	[36]
*O. europaea* var. Chemlali North	not performed	- Olive leaf extract (1:50 w/v). - pH = 2 by adding HCl 6M. - addition of pepsin (0.02 g/g sample, 975 U/mg protein) from porcine stomach mucosa. - incubation at 37 °C for 2 h in a shaking water bath.	- pH = 6.5 by adding NaHCO_3_ 1M - addition of pancreatin-bile mixture (0.05 g pancreatin (activity equivalent to 4X USP specifications) and 0.31 g salts in 12.5 mL of NaHCO_3_ 0.1M) - incubation at 37 °C for 2 h in a shaking water bath. - samples were frozen to stop reaction.	[37]
*O. europaea* var. Carrasqueña	- Olive leaf extracts (1 g). - addition of oral fluid which contained 1 mL of human saliva/g extract. - Incubation at 37 °C for 20 s.	- addition of SGF (3.6 mL, containing 0.2 g pepsin and 0.125 g NaCl in deionized water). - pH = 2.2. - incubation at 37 °C for 20 min.	- pH = 6.5 by adding NaOH. - addition of SIF (3.6 mL, containing pancreatin (20 mg), lipase (5 mg), cholic acid (10 mM), and deoxycholic acid (10 mM) in PBS buffer 0.1 M). - incubation at 37 °C for 20 min in a shaking water bath.	[38]
*O. europaea* var. Coratina	Novel food: “Taralli”, enriched with olive leaf extract (obtained with ethanol/water, 50:50 *v/v*) - addition of oral phase solution (6 mL, containing 5% mucin, 3% uric acid, 40% urea, and 10.6 g of α-amylase). - incubation at 37 °C for 10 min (85 rpm). - dilution of samples to 30 mL with 0.9% NaCl.	- addition of porcine pepsin solution (2 mL, 20 mg/mL in HCl 0.1 M). - pH = 3.0 by adding HCl 0.1 M. - incubation at 37 °C for 1 h in a covered shaking water bath (85 rpm). - addition of 0.9% saline solution.	- pH = 6.5 by adding NaHCO_3_ 1.0 M. - addition of intestinal enzymes (2.0 mL containing 30 mg/mL pancreatin, 15 mg/mL lipase in NaHCO_3_ 0.1 M) and porcine bile salts (3 mL, 120 mg/mL bile extract in NaHCO_3_ 0.1 M). - addition of 0.9% saline solution. - incubation at 37 °C for 2 h in a shaking water bath. - acidification with 2% aqueous acetic acid (1:1).	[39]
**Residues from Olive Oil Process**				
Olive mill wastewater (OMWW)	not performed	Purified phenolic fractions from OMWW extracts (3 mL) diluted with NaCl 0.9% at pH 7. - addition of porcine pepsin solution (0.9 mL, 40 mg/mL in HCl 0.1 N). - pH = 2.5 by adding HCl 1 N - incubation at 37 °C for 1 h in a covered shaking water bath.	- pH = 5.3 by adding NaHCO_3_ 100 mM/NaOH 1N. - addition of small intestinal enzyme solution (2.7 mL of porcine lipase (2 mg/mL), pancreatin (4 mg/mL), and bile (24 mg/mL) in NaHCO_3_ (100 mM). - pH = 6.5 ± 0.1 by adding NaOH 1N. - incubation at 37 °C for 1 h in a covered shaking water bath.	[40]
Freeze-dried olive cake extracts from *O. europaea* var. Arbequina	- Extracts (1.5 g). - addition of amylase in phosphate buffer solution. - Incubation for 5 min.	- pH = 2 by adding HCl concentrate. - addition of porcine pepsin solution. - Incubation for at 37 °C for 2 h.	- addition of bile salts (2.5 mL) and pancreatin (2.5 mL) (8 g/L). - pH = 6.5 by adding NaHCO_3_. - A continuous-flow dialyzed step was chosen keeping the temperature constant, under 37 °C.	[9]
Liquid-enriched olive pomace powder (LOPP) from *O. europaea* var. Galega Vulgar	- LOPP extracts - addition of α-amylase (0.6 mL, 100 U/mL). - incubation at 37 °C for 1 min and 200 rpm.	- pH = 2 by adding HCl 1M. - addition of pepsin solution (25 mg/mL) from porcine stomach mucosa at a rate of 0.05 mL/mL sample. - incubation at 37 °C for 1 h in a shaking bath.	- pH = 6.0 by adding NaHCO_3_ 1M. - addition of pancreatin (2 g/L, from porcine pancreas 2g/L) and bile salts (12 g/L) at a ratio of 0.25 mL/mL sample. - incubation at 37 °C for 2 h in a shaking bath. - Dialysis process: dialysis tubing (3.5 kDa molecular weight cut-off) filled with NaHCO_3_ 1 M). - Incubation at 37 °C in a shaking water bath (50 rpm) for 2 h.	[41]
Olive pomace extracts (OPE)	- OPE samples (200 mg alone or mixed with 600 mg of food matrix). - addition of SSF (1.9 or 2.3 mL). - α-amylase (1500 U/mL). - Incubation at 37 °C for 2 min.	- addition of SGF (2.5 mL) containing pepsin (25,000 U/mL). - pH = 3. - incubation at 37 °C for 2 h.	- addition of SIF (5 mL) containing bile salts and pancreatin (800 U/mL). - pH = 7. - incubation at 37 °C for 2 h.	[11]

SSF, simulated salivary fluid; SGF, simulated gastric fluid; SIF, simulated intestinal fluid; EVOO, extra virgin olive oil.

**Table 2:** Total phenolic content determined by the Folin–Ciocalteu method in the oil extracts and in the bioaccessible and bioavailable fractions after in-vitro digestion of the oil samples.

Total Phenolic Content					
	Oil Extract	Bioaccessible Fraction	Bioavailable Fraction	Residual Fraction	References
	**mg Ty/kg**	**mg Ty/kg**			[5]
EVOO 1 (Oliarola del Bradano)	291 ± 8	276 ± 8	-	-	
EVOO 2 (Oliarola del Bradano)	578 ± 9	583 ± 9	-	-	
EVOO 3 (Oliarola del Bradano)	538 ± 8	551 ± 14	-	-	
EVOO 4 (Oliarola del Bradano)	663 ± 9	655 ± 9	-	-	
EVOO 5 (Maiatica)	277 ± 6	262 ± 14	-	-	
EVOO 6 (Maiatica)	286 ± 11	262 ± 15	-	-	
EVOO 7 (Coratina)	451 ± 11	437 ± 15	-	-	
EVOO 8 (Coratina)	473 ± 15	483 ± 8	-	-	
EVOO 9 (Coratina)	713 ± 13	724 ± 9	-	-	
EVOO 10 (Coratina)	354 ± 15	376 ± 8	-	-	
	**mg CAE/kg**	**mg CAE/kg**			[10]
EVOO 11 (Cornicabra)	317	891	-	-	
EVOO 12 (Picual)	256	630	-	-	
EVOO 13 (Manzanilla)	234	685	-	-	
EVOO 14 (Picudo)	207	764	-	-	
EVOO 15 (Hojiblanca)	169	689	-	-	
EVOO 16 (Arbequina)	153	613	-	-	
	**mg GA/kg**	**mg GA/kg**	**Absorption** (% from the Initial Solution) *	**mg GA/kg**	[12]
EVOO 17 (Picual)	368 ± 32	1029 ± 221	25.41 ± 5.07	10.38	
	**mg CAE/kg**	**mg CAE/kg**		**mg CAE/kg**	[17]
EVOO 18 (Arbequina, Granada)	168 ± 1.14	451 ± 29.8	49.1 ± 4.74	110 ± 12.7	
EVOO 19 (Arbequina, Jaén)	163 ± 13.519	538 ± 53.1	55.9 ± 5.88	101 ± 25.1	
EVOO 20 (Arbequina, Málaga)	302 ± 29.45	506 ± 3.06	37.9 ± 7.00	140 ± 16.2b	
EVOO 21 (Arbequina, Cádiz)	196 ± 13.5	392 ± 66.1	44.5 ± 3.04	67.7 ± 24.8	
EVOO 22 (Arbequina, Sevilla)	227 ± 9.27	411 ± 48.0	102 ± 19.4	194 ± 33.6	
EVOO 23 (Arbequina, Albacete)	197 ± 13.5	566 ± 38.9	32.5 ± 3.57	127 ± 41.2	
EVOO 24 (Arbequina, Toledo)	174 ± 2.85	436 ± 4.02	87.7 ± 7.86	109 ± 12.2	
EVOO 25 (Arbequina, Valladolid)	290 ± 15.61	432 ± 18.9	110 ± 17.4	136 ± 15.7	
EVOO 26 (Arbequina, Lérida)	104 ± 21.3	391 ± 81.1	83.4 ± 9.73	96.8 ± 16.1	
EVOO 27 (Arbequina, Rio Grande do Sul)	151 ± 4.44	531 ± 12.1	97.0 ± 15.4	121 ± 9.21	
EVOO 28 (Arbequina, Minas Gerais)	75.0 ± 2.18	473 ± 41.6	97.6 ± 21.0	92.6 ± 4.83	
	**mg CAE/kg**	**mg CAE/kg**		%	[32]
EVOO 29 (Hojiblanca, 2014)	397	1031	-	18–52%	
EVOO 30 (Hojiblanca, 2014)	367	633	-	18–52%	
EVOO 31 (Hojiblanca, 2015)	394	1018	-	18–52%	
EVOO 32 (Hojiblanca, 2015)	405	893	-	18–52%	
EVOO 33 (Arbequina, 2014)	231	371	-	18–52%	
EVOO 34 (Arbequina, 2014)	222	451	-	18–52%	
EVOO 35 (Arbequina, 2015)	230	1453	-	18–52%	
EVOO 36 (Arbequina, 2015)	226	1347	-	18–52%	

Ty, tyrosol equivalents; CAE, caffeic acid equivalents; GA, gallic acid equivalents. * Absorption of TPC (% from the initial solution) across Caco-2 monolayers after 2 h of incubation with the bioaccessible fractions of the oil samples. In Borges et al. [17], absorption was calculated as the percentage absorbed in the basal chamber of the initial quantity in the apical chamber.

**Table 3:** Antioxidant capacity measured by the DPPH assay in the oil extracts and in the bioaccessible and bioavailable fractions after in-vitro digestion of the oil samples.

Antioxidant Capacity					
	Oil Extract	Bioaccessible Fraction	Bioavailable Fraction Absorption (% from the Initial Solution) *	Residual Fraction	References
	**mmol Trolox/kg**	**mmol Trolox/kg**			[10]
EVOO 11 (Cornicabra)	0.60 ± 0.13	2.51 ± 0.90	-	-	
EVOO 12 (Picual)	0.40 ± 0.13	0.95 ± 0.24	-	-	
EVOO 13 (Manzanilla)	0.60 ± 0.09	1.95 ± 0.24	-	-	
EVOO 14 (Picudo)	0.62 ± 0.13	1.55 ± 0.30	-	-	
EVOO 15 (Hojiblanca)	0.36 ± 0.13	1.08 ± 0.56	-	-	
EVOO 16 (Arbequina)	0.44 ± 0.12	0.84 ± 0.39	-	-	
	**mmol Trolox/kg**	**mmol Trolox/kg**		%	[12]
EVOO 17 (Picual)	0.65 ± 0.001	0.88 ± 0.01	2.27 ± 0.22	6.7	
	**mmol Trolox/kg**	**mmol Trolox/kg**		**mmol Trolox/kg**	[17]
EVOO 18 (Arbequina, Granada)	0.93 ± 0.05	2.58 ± 0.31	50.1 ± 2.21	0.38 ± 0.07	
EVOO 19 (Arbequina, Jaén)	1.45 ± 0.18	2.27 ± 0.45	43.0 ± 0.70	0.42 ± 0.02	
EVOO 20 (Arbequina, Málaga)	1.58 ± 0.01	0.99 ± 0.08	52.4 ± 12.8	-	
EVOO 21 (Arbequina, Cádiz)	0.52 ± 0.02	2.54 ± 0.13	30.7 ± 0.10	0.41 ± 0.04	
EVOO 22 (Arbequina, Sevilla)	1.22 ± 0.30	2.08 ± 0.27	40.0 ± 2.22	-	
EVOO 23 (Arbequina, Albacete)	0.74 ± 0.05	2.56 ± 0.19	39.2 ± 5.98	0.18 ± 0.18	
EVOO 24 (Arbequina, Toledo)	0.81 ± 0.03	1.91 ± 0.24	52.2 ± 4.54	0.29 ± 0.04	
EVOO 25 (Arbequina, Valladolid)	1.52 ± 0.04	2.33 ± 0.12	38.9 ± 4.76	-	
EVOO 26 (Arbequina, Lérida)	0.65 ± 0.20	2.51 ± 0.07	44.4 ± 6.74	0.35 ± 0.04	
EVOO 27 (Arbequina, Rio Grande doSul)	0.97 ± 0.01	2.97 ± 0.26	51.5 ± 5.56	-	
EVOO 18 (Arbequina, Minas Gerais)	0.56 ± 0.06	50.0 ± 3.64	50.0 ± 3.64	-	
	**mmol Trolox/kg**	**mmol Trolox/kg**			[32]
EVOO 29 (Hojiblanca, 2014)	0.67	0.98	-	-	
EVOO 30 (Hojiblanca, 2014)	0.70	0.93	-	-	
EVOO 31 (Hojiblanca, 2015)	1.39	4.97	-	-	
EVOO 32 (Hojiblanca, 2015)	1.41	3.83	-	-	
EVOO 33 (Arbequina, 2014)	1.52	2.28	-	-	
EVOO 34 (Arbequina, 2014)	0.93	1.88	-	-	
EVOO 35 (Arbequina, 2015)	0.70	5.44	-	-	
EVOO 36 (Arbequina, 2015)	0.71	4.83	-	-	

* Absorption of TPC (% from the initial solution) across Caco-2 monolayers after 2 h of incubation with the bioaccessible fractions of the oil samples. In Borges et al. [17], absorption was calculated as the percentage absorbed in the basal chamber of the initial quantity in the apical chamber.

## Data Availability

Data sharing not applicable.

## Abbreviations

AB-BD	after biosorption-before digestion
AB-AD	after biosorption-after digestion
AC	antioxidant capacity
AP	apical
Api	apigenin
BB-AD	before biosorption-after digestion
BB-BD	before biosorption-before digestion
BdC	Bella di Cerignola
BL	basolateral
CA	caffeic acid
CAE	caffeic acid equivalents
CEL	Cellina di Nardò
COM	comselogoside
CoQ10	coenzyme Q10.
DAD	diode array detection
DPPH	2,2′-diphenyl-1-picrylhydrazyl radical
dw	dry weight
EFSA	European Food Safety Agency
ESI	Electrospray ionization
EU	European Union
EVOO	extra virgin olive oil
FaSSGF	fasted-state-simulated gastric fluid
FaSSIF	fasted-state-simulated intestinal fluid
FeSSGF	fed-state-simulated gastric fluid
FeSSIF	fed-state-simulated intestinal fluid
FD	freeze-drying
FLD	fluorescence detection
FW	fresh weight
GAE	gallic acid equivalents
HAD	hot air drying
HPLC	high-performance liquid chromatography
HR	high resolution
HTy	hydroxytyrosol
HTy-Ac	hydroxytyrosol acetate
IN	inulin
isoVB	iso verbascoside
Lig	ligstroside
LOPP	liquid-enriched olive pomace powder
Lut	luteolin
MD	maltodextrin
MedDiet	Mediterranean diet
MS	mass spectrometry
Ole	oleuropein
OMWW	olive mill waste water
OP	olive pomace
OPE	olive pomace extracts
QqTOF	tandem quadrupole/time-of-flight
QTOF	quadrupole time-of-flight
SEC	caffeoyl-6′-secologanoside
SGD	simulation of gastrointestinal digestion
SGF	simulated gastric fluid
SIF	simulated intestinal fluid
SSF	simulated salivary fluid
TAGs	triacylglycerides
TDB	Termite di Bitetto
TIPC	total individual phenolic content
TOF	time-of-flight
TPC	total phenolic content
Ty	tyrosol
UHPLC	ultra-high-performance liquid chromatography
UHR	ultra-high resolution
US	ultrasound-assisted
UV-VIS	ultraviolet-visible
VB	verbascoside
VOO	virgin olive oil
WoS	Web of Science
